# Unravelling the origin of the basket stars and their allies (Echinodermata, Ophiuroidea, Euryalida)

**DOI:** 10.1038/s41598-018-26877-5

**Published:** 2018-05-31

**Authors:** Ben Thuy, Sabine Stöhr

**Affiliations:** 1 Natural History Museum Luxembourg, Department of Palaeontology, 25 rue Münster, 2160 Luxembourg City, Luxembourg; 2 Swedish Museum of Natural History, Box 50007, 10405 Stockholm, Sweden

## Abstract

Euryalids, which include the spectacular basket stars, form a morphologically aberrant group of brittle stars. Surprisingly, the most recent molecular work found them to be sister to ophiurid brittle stars, thus challenging the traditional dichotomy between euryalids and non-euryalids, and leaving an enormous ghost lineage of more than 100 million years between the oldest unambiguous euryalid fossils and their predicted divergence from ophiurids during the Triassic. Here we examine both previously known and newly collected fossils to explore the evolutionary history of euryalids. A morphology-based phylogenetic estimate confirms the Triassic *Aspiduriella* as a basal member of the euryalid clade that superficially resembles members of the living ophiurid sister clades. Furthermore, we use lateral arm plates and vertebrae to identify two new Jurassic ophiuroids, *Melusinaster alissawhitegluzae* and *Melusinaster arcusinimicus*, as early euryalids that are morphologically intermediate between *Aspiduriella* and extant euryalids. Our phylogenetic analysis is the first to combine data from completely preserved skeletons and from microfossils in order to bridge morphological and stratigraphical gaps between the sampled taxa. It fills a major gap in the fossil record of euryalids and sets a robust phylogenetic framework to understand the morphological transition from ophiurid-like ancestors to the typical modern euryalids better.

## Introduction

Euryalids (order Euryalida) undoubtedly rank among the most extraordinary of living brittle stars. They include the basket stars of the families Gorgonocephalidae (“Gorgon Heads”) and Euryalidae that unfold their bewilderingly complex array of branched arms to suspension feed at night. Other Euryalida (also known as snake stars) have extremely long, unbranched arms that are capable of tight coiling, and use these to cling to octocorals and other hosts. Morphologically, Euryalida differ so markedly from other ophiuroids^1,2^ that, in the past, they were considered a group of their own^3–7^, prompting the subdivision of ophiuroids into euryalids on the one hand and non-euryalids on the other.

As clear-cut as this distinction seemed, it caused considerable controversy and perplexity with respect to its evolutionary origin. Attempts to identify transitional euryalid morphologies in non-euryalid ophiuroids, in particular the Ophiomyxidae^8,9^ turned out to be stopgap solutions at best^10^. For a long time, palaeontological evidence was not helpful either. Until recently, only few unambiguous euryalid fossils were known, dating from strata as young as latest Cretaceous and Neogene^11,12^ and thus leaving a ghost lineage of more than 100 million years to the assumed basal divergence between euryalids and non-euryalids. To make matters worse, the Paleozoic *Onychaster* Meek and Worthen, 1868, long considered a euryalid ancestor, has recently been debunked as a stem-group lookalike that lacks any closer affinities with euryalids or other modern ophiuroids^13^.

The most recent advances in ophiuroid systematics and phylogeny^10,14–16^ have fundamentally changed perspectives by unveiling unexpectedly close ties between euryalid and ophiurid brittle stars, forming a clade that is sister to all remaining modern ophiuroids. However, these new insights have still implied an excessively long missing fossil record between the predicted euryalid divergence during the Triassic and the oldest unambiguous euryalid fossils from the uppermost Cretaceous, but for the first time, it has become clear that the assumed early euryalids would have had ophiurid-like morphologies^14^. In parallel, recent advances in ophiuroid studies have fleshed out the systematic significance of previously underexplored micro-structural features to reconstruct phylogenetic relationships between ophiuroid groups^17^ and furthermore have shown that ophiuroid microfossil evidence can be integrated into phylogenetic estimates^15^.

With these promising new approaches in hand, we set out to re-assess the ophiuroid fossil record in order to explore euryalid origins and in particular to identify stem members and transitional fossils that would bridge the extreme morphological disparity with living sister clades. Here we provide detailed descriptions of both previously known and newly collected fossils, in particular belonging to the Triassic group of *Aspiduriella* and to a new Jurassic genus, and put these in a phylogenetic context to explore the evolutionary history of euryalids.

## Material and Methods

### Specimen treatment

Most of the fossil specimens described herein were provided by private collector Manfred Kutscher (Saßnitz, Germany) and comprise *Aspiduriella streichani* (Kutscher, 1987) from the Middle Triassic (Anisian, ca. 244 million years) type locality of the species at Rüdersdorf near Berlin (Germany), *Aspiduriella scutellata* (Blumenbach, 1804) from the Middle Triassic (atavus Zone, Anisian, ca. 242 million years) of Schillingstadt and Wollmershausen and the Middle Triassic (praenodosus to nodosus Zone, Ladinian, ca. 240 million years) of Bettenfeld, all in southwestern Germany, as well as specimens of *Melusinaster alissawhitegluzae* gen. et sp. nov. as part of the original material from the Lower Jurassic (Aalensis Zone, Toarcian, ca. 174 million years) of Quedlinburg, eastern Germany, as described in Kutscher^18^. Specimens of *Melusinaster arcusinimicus* gen. et sp. nov. were picked from micropalaeontological samples from the Middle Jurassic (Humphriesianum Zone, Bajocian, ca. 167 million years) of southern Luxembourg.

Lateral arm plates of Recent ophiuroids were extracted from proximal arm portions macerated in household bleach and then rinsed in tap water following the method described by Thuy and Stöhr^17^. Selected fossils and isolated lateral arm plates of extant taxa were mounted on aluminium stubs and gold-coated for scanning electron microscopy (SEM) using a Jeol Neoscope JCM-5000 and a Hitachi FE-SEM 4300. Type and illustrated specimens were deposited in the collections of the Swedish Museum of Natural History (SMNH), the Natural History Museum Luxembourg (MnhnL) and the Geoscientific Museum of the Georg-August-University Göttingen, Germany (GZG.INV.).

### Phylogeny

In order to explore the phylogenetic position of the fossils described herein with respect to modern euryalids and ophiurids, we performed a Bayesian inference analysis using the matrix elaborated by Thuy and Stöhr^15^ comprising 41 Recent and four ingroup taxa, and *Aganaster gregarius* (Meek and Worthen, 1869) as outgroup taxon, adding *Aspiduriella scutellata* and *Melusinaster alissawhitegluzae* gen. et sp. nov., both type species of their respective genera. Furthermore, we added *Aspiduriella streichani* because it had previously been identified as a possible member of the euryalid stem lineage^14^.

Character scoring was performed as outlined by Thuy and Stöhr^15^ using the same list of characters (and character acronyms), with the following exceptions as a result of ongoing improvement of our ophiuroid character matrix (see Supplementary Data 1 and 2 and Supplementary Fig. 1 for details). In character D-RS-6, the states were modified in order to differentiate between taxa with an incised outline of the radial shields and taxa with extended radial shield contours; the character was thus scored as D-RS-6 radial shield abradial edge: without incisions or extensions (0), with extensions (regular outline enlarged, Supplementary Fig. 1c) (1), with incisions (regular outline carved, Supplementary Fig. 1a,b) (2). In GP-2, the states were modified to describe the shape variation in abradial genital plates better; the character was scored as GP-2 abradial genital plate: paddle-shaped (Supplementary Fig. 1j) (0), sabre-like with longitudinal rim (Supplementary Fig. 1f) (1), sabre-like with widened distal portion (Supplementary Fig. 1g) (2), sabre-like without ridge or rim (Supplementary Fig. 1h) (3), half-ring shaped (Supplementary Fig. 1i) (4), scale-like, wide, with central longitudinal ridge (Supplementary Fig. 1e) (5). In LAP-SA-20, the states were modified to record the morphological variety of spine articulations within the Euryophiurida (plus the outgroup) better; the character was thus scored as LAP-SA-20 when dorsal and ventral lobes absent: muscle opening encompassed by poorly defined circular elevation (Supplementary Fig. 1k) (0), simple stereom and/or vertical ridge distally and wavy ridge proximally (Supplementary Fig. 1l) (1), vertical mouth-shaped, sharply defined elevation (Supplementary Fig. 1m) (2). Furthermore, the following character was added: GP-10 conspicuous perforation on abradial genital plate: absent (0), present (Supplementary Fig. 1f) (1).

Bayesian inference analysis was performed using MrBayes^19^ using the same parameters, settings and prior assumptions as in Thuy and Stöhr^15^. We assume a priori that during the long evolutionary history of the euryalids, characters have been subjected to variable rates of evolution, and we accounted for missing data in our matrix. The effects of these assumptions were tested in our previous study^15^ and found to give the most likely results, confirmed also by molecular data^14,16^. Wright & Hillis^20^ have shown that likelihood-based methods such as Bayesian statistics outperform parsimony under these circumstances and produce more reliable tree hypotheses. Each of the resulting trees receives a likelihood score and a majority rule consensus tree is calculated to summarize the variation across the trees. The likelihood of the tree being true is given as the posterior probability, which is the probability of the event after taking the evidence into account. Average standard deviations of split frequencies stabilized at about 0.007–0.01 after 3 million generations (mgen), sampled every 1,000 generations. The first 25% of the trees were discarded as burnin. The consensus trees were examined with the software FigTree v. 1.4.2 by Rambaut (http://tree.bio.ed.ac.uk/software/figtree/). As is common standard in statistics, we regard confidence intervals of 95–99% as strong support for a node to be true, and at least 90% probability as good support.

In order to explore the position of *Ophiosparte gigas* Koehler, 1922 within the euryophiurids, we performed an additional analysis in which we considered some of the lateral arm plate character states of *O. gigas* to resemble secondarily the states found in euryalids. Specifically, we scored the characters LAP-O-2, LAP-O-8 and LAP-PE-3 as in *Ophiura ophiura* (Linnaeus, 1758), assuming that they are secondarily modified variants of these states rather than primary equivalents of euryalid states. In a final step, we ran an analysis without *Ophiosparte gigas*.

### Nomenclatural acts

The electronic edition of this article conforms to the requirements of the amended International Code of Zoological Nomenclature, and hence the new names contained herein are available under that Code from the electronic edition of this article. This published work and the nomenclatural acts it contains have been registered in ZooBank, the online registration system for the ICZN. The ZooBank LSIDs (Life Science Identifiers) can be resolved and the associated information viewed through any standard web browser by appending the LSID to the prefix “http://zoobank.org/”. The LSID for this publication is: urn:lsid:zoobank.org:pub:2949CE4E-F6C4-491B-B393-F375D6EB94E3; for *Melusinaster*: urn:lsid:zoobank.org:act:C70BEA51-C832-4B88-9A7D-310370DC8D00; for *Melusinaster alissawhitegluzae*: urn:lsid:zoobank.org:act:AA407FFB-24B0-4781-A336-79D9A092EA1A; for *Melusinaster arcusinimicus*: urn:lsid:zoobank.org:act:51A6B3A2-73A2-4B9E-A5D9-B01A6FE500B7. The electronic edition of this work was published in a journal with an ISSN, and has been archived and is available from the following digital repositories: PubMed Central, LOCKSS.

## Results

### Phylogenetic relationships

The initial Bayesian estimate of the updated matrix of Thuy and Stöhr^15^ including *Aspiduriella scutellata*, *A. streichani* and *Melusinaster alissawhitegluzae* gen. et sp. nov. produced a well-resolved tree with reasonably high support values. Tree topology largely followed the results obtained by Thuy and Stöhr^15^, with few exceptions pertaining to basal nodes. *Aspiduriella scutellata*, *A. streichani* and *Melusinaster alissawhitegluzae* gen. et sp. nov. formed a robust clade in a poorly supported sister relationship with the euryophiurids (except for *Ophiomusa lymani*) (Supplementary Fig. 2).

Re-scoring of *Ophiosparte gigas* in order to differentiate between primary absence and secondary loss of lateral arm plate ornamentation fundamentally changed the topology within the euryophiurid clade (still excluding *Ophiomusa*). The euryalids are in a weakly supported sister relationship with the clade of *Aspiduriella* and *Melusinaster* and, together, are sister to the ophiurids including *Ophiosparte* (Supplementary Fig. 3).

**Figure 1:**
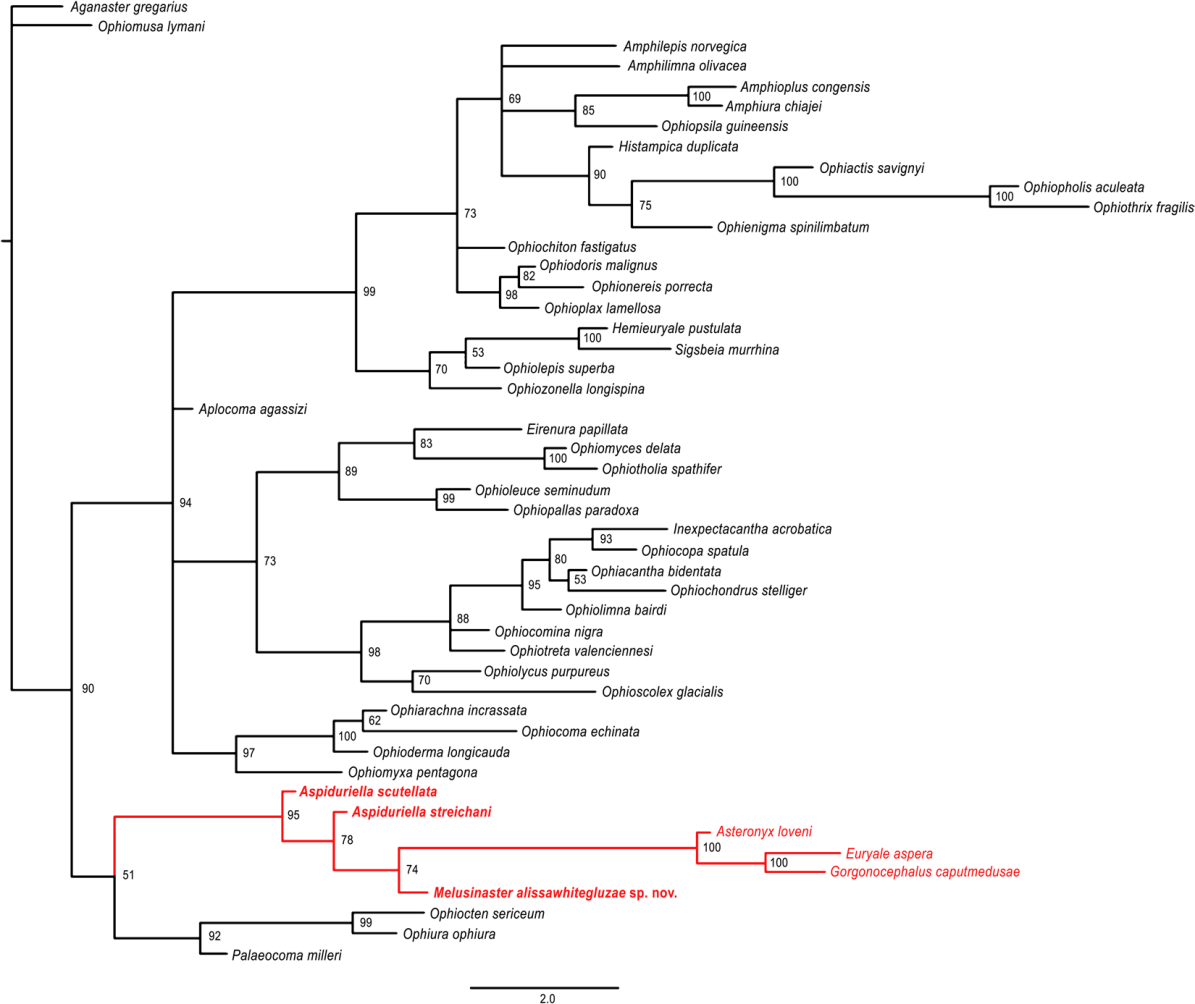
Phylogenetic tree of the updated matrix of Thuy and Stöhr^15^ including the extinct species *Aspiduriella scutellata*, *A*. *Streichani* and *Melusinaster alissawhitegluzae* gen. et sp. nov. (marked in bold) but excluding *Ophiosparte gigas*, inferred using MrBayes. Numbers at nodes indicate posterior probabilities. Extinct species are marked by a cross.

**Figure 2:**
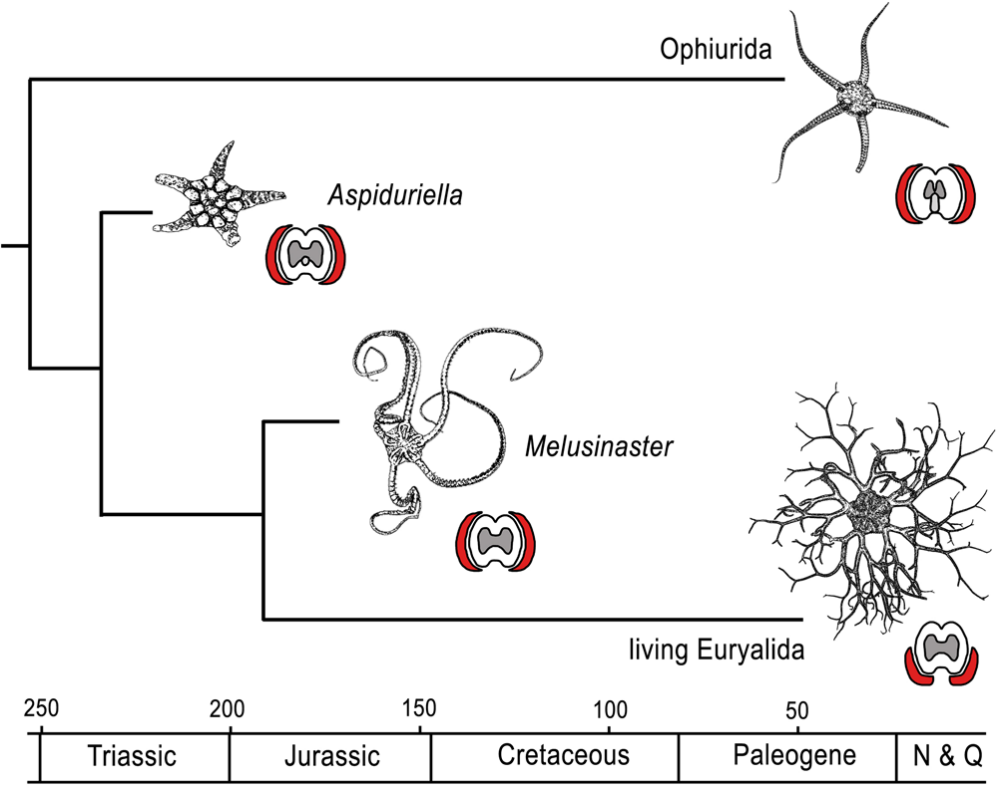
Evolutionary tree of euryalid ophiuroids based on the phylogenetic estimate of Fig. 1. Sketches show typical representatives of relevant taxa and a hypothetic reconstruction of *Melusinaster* gen. nov. based on morphologically similar modern euryalids. Sketches of arm sections show distal vertebral articulations with zygocondyles in dark grey and zygosphene in light grey, and the position of the lateral arm plates in red. N & Q means Neogene and Quaternary, numbers indicate time scale in million years before present.

The exclusion of *Ophiosparte* again changes the topology within the euryophiurids (minus *Ophiomusa*): euryalids form a robust clade together with a nested succession of *Aspiduriella scutellata*, *A. streichani* and *Melusinaster alissawhitegluzae* gen. et sp. nov. at their base (Figs 1 and 2). The ophiurids form another robust clade in a poorly supported sister relationship with the euryalid clade.

### Systematic palaeontology

Class Ophiuroidea Gray, 1840

Subclass Myophiuroidea Matsumoto, 1915

Infraclass Metophiurida Matsumoto, 1913 (crown-group of Ophiuroidea)

Superorder Euryophiurida O’Hara, Hugall, Thuy, Stöhr and Martynov, 2017

Order Euryalida Lamarck, 1816

Family unknown

Genus *Aspiduriella* Bolette, 1998

Type species: *Ophiura loricata* Goldfuss, 1833, junior synonym of *Aspiduriella scutellata* (Blumenbach, 1804) (see Boehm^21^).

#### Diagnosis

Small to medium-sized ophiuroids with thick and tumid general plating except for a few thin disc scales; dorsal disc covered by conspicuously large radial shields forming continuous or at least near-continuous ring enclosing granule-covered primary plates; adoral shields meeting over their entire length at proximal tip of oral shield; second oral tentacle pores entering mouth slit via shallow embayment; teeth and oral papillae small; arms short and rapidly tapering; lateral arm plates tumid, crescent-shaped, devoid of outer surface ornamentation except for poorly defined spurs on proximal edge; spine articulations with muscle opening bordered by poorly defined, arched ridge proximally, and by thick lip-shaped ridge distally; distal articulation face of vertebrae with tiny zygosphene between two large, parallel zygocondyles.

Aspiduriella scutellata (Blumenbach, 1804)

Figure 3

*1804 *Asterites scutellatus* Blumenbach, p. 24, pl. 2, fig. 10.

1833 *Ophiura loricata* Goldfuss, p. 192, pl. 62, fig. 7a–c.

1883 *Aspidura loricata* (Goldfuss): Zittel, p. 449, fig. 321a–c.

1889 *Aspidura loricata* (Goldfuss): Boehm, p. 283.

1928 *Aspidura scutellata* (Blumenbach): Schmidt, p. 130, fig. 250a–b.

1940 *Aspidura scutellata* (Blumenbach): Kutscher, p. 13, pl. 1, Fig. 3.

1966 *Aspidura loricata* (Goldfuss): Spencer & Wright, p. U95, fig. 81.5a-b.

1988 *Aspidura scutellata* (Blumenbach): Calzada & Gutiérrez, p. 32, Fig. 1.

1998 *Aspiduriella scutellata* (Blumenbach): Bolette, p. 401.

2002 *Aspiduriella scutellata* (Blumenbach): Radwański, p. 405.

Material examined: GZG.INV.90012 - GZG.INV.90015.

#### Description

**Figure 3:**
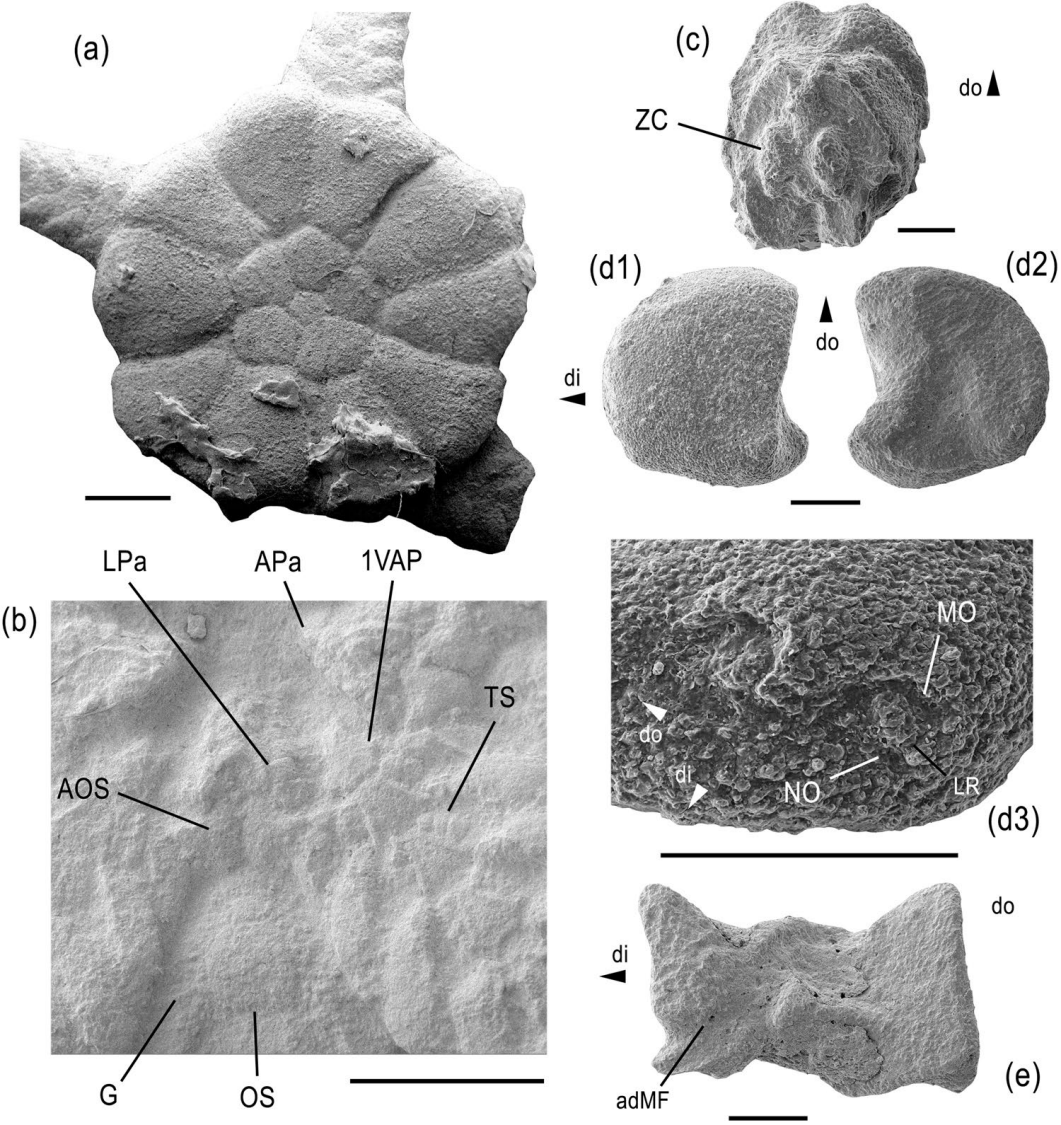
*Aspiduriella scutellata* from the Middle Triassic of Wollmershausen (**a**,**b**), Bettenfeld, Bavaria (**c**), and Schillingstadt (**d**,**e**), southwestern Germany. (**a**) GZG.INV.90012, articulated disc preserving proximal to median arm segments in dorsal view. (**b**) Detail of GZG.INV.90012 showing mouth plating. (**c**) GZG.INV.90013 isolated vertebra in dorso-distal view. (**d**) GZG.INV.90014 isolated proximal lateral arm plate in external (d1) and internal (d2) views and with detail of spine articulations (d3). (**e**) GZG.INV.90015 isolated oral plate in adradial view. Abbreviations: adMF: adradial muscle fossa; AOS: adoral shield; APa: apical oral papilla; di: distal; do: dorsal; G: granule; LPa: lateral oral papilla; LR: lip-shaped ridge; MO: muscle opening; NO: nerve opening; OS: oral shield; TS: tentacle scale; ZC: zygocondyle; 1VAP: first ventral arm plate. Scale bars equal 1 mm (**a**,**b**) and 0.2 mm (**c**–**e**).

Small species (disc diameter of examined specimens around 5 mm), disc round, dorsal side almost entirely covered by continuous ring of radial shields and primary plates; radial shields conspicuously large and thick, accounting for more than half of disc radius, not covered by granules, rounded isosceles triangular with very weakly concave lateral edges, with distal edge thickened and projecting ventralwards; ring of radial shields enclosing central and radial primary plates all of nearly equal size and with plate boundaries covered by tiny granules; only few other, tiny scales discernible between primary plates and radial shields. Ventral disc interradii covered by small, rounded hexagonal oral shield, almost as wide as long, proximally and laterally bordered by much smaller scales densely covered by granules (Fig. 3b), and distally bordered by a nearly equal-sized and similarly rounded pentagonal plate; genital slits extending from adoral shields to almost half of the interradius, no appendages other than regular disc granules; adoral shields short, slightly longer than wide, lozenge-shaped, meeting over their entire length (Fig. 3b), boundaries covered by tiny granules; jaws relatively short and slender, pointed; dissociated oral plate longer than high (Fig. 3e); adradial muscle fossa lining ventro-distal edge of articulation area; six small, lateral oral papillae forming a continuous row, gradually increasing in size from the jaw tip to the adoral shield and at the same time gradually changing from pointed to block-like (Fig. 3b); second oral tentacle pore entering mouth slit via shallow embayment; first ventral arm plate triangular with proximal tip projecting into mouth slit and lateral edges bearing two to three block-like papillae similar in shape and size to median lateral oral papillae; single, very small, conical, pointed apical oral papilla.

Arms short, broad at base, rapidly tapering (Fig. 3a); not branching and not covered by granules except for the plate boundaries on the ventral side of the proximalmost three segments; dorsal arm plates small, fan-shaped, separated by lateral arm plates on all arm segments; lateral arm plates (Fig. 3d) thick, bulging, crescent-shaped, with pointed dorso- and ventro-proximal tips; outer surface stereom with simple trabecular intersections, devoid of any ornamentation except for a poorly defined, weakly prominent spur in the ventral third of the proximal edge, dorsally bordered by a slightly sunken central part of the outer surface; three to four spine articulations sunken into distal edge of lateral arm plates, equal-sized, with dorsalwards increasing distance between the spine articulations (Fig. 3d); muscle opening of spine articulations proximally bordered by arched, non-denticulate vertical ridge, and distally separated from nearly equal-sized nerve opening by large, thick, lip-shaped and strongly prominent ridge; inner side of lateral arm plates with single, vertical ridge composed of the same stereom as remaining inner side and with a kink between its dorso-proximalwards pointing dorsal portion and its ventro-proximalwards pointing ventral portion (Supplementary Fig. 4a); arm spines parallel to the arm axis, shorter than half the length of an arm segment, nearly equal-sized, smooth, laterally flattened and with blunt tip; no hooks or modified spines discernible; ventral arm plates small, in contact only on proximalmost arm segments, nearly diamond-shaped with obtuse distal angle, weakly concave latero-proximal edges lined by a ridge, and acute proximal angle; tentacle openings large, developed as elongate between-plate openings at an angle to the arm axis, covered by at least six flat, elongate tentacle scales, three at the lateral arm plate and another three at the ventral arm plate; vertebrae (Fig. 3c) compact with small, non-projecting muscle fossae and a single very large lateral articulation ridge for the lateral arm plate; tiny zygosphene between two large, parallel and almost hourglass-shaped zygocondyles.

#### Remarks

Preliminary observations on possible basal euryalid affinities of the genus *Aspiduriella* focused on the species *A. streichani* because it was found to display the relevant characters in the most compelling way^14,22^. For the present, more exhaustive study, we decided to include *Aspiduriella scutellata* because it is the type species of the genus and thus allows for a generalised conclusion on the systematic position of the genus irrespective of whether other assumed congeners follow the pattern.

*Aspiduriella scutellata* was the first fossil ophiuroid ever to have been described^23^ and has since been revised and illustrated by numerous authors. It was subjected to several genus-level transfers and synonymized with *Ophiura loricata*, as shown by our representative but non-exhaustive synonymy. Yet, despite considerable attention that the species has received over the past two centuries, knowledge of its morphology has remained surprisingly superficial. As a result, few studies ventured to discuss the systematic position of the *Aspiduriella* group in earnest^24^.

Our re-assessment aimed at unlocking microstructural features of phylogenetic value^15^ and successfully allowed the inclusion of *A. scutellata* in a morphology-based phylogenetic analysis in order to elucidate its position within ophiuroid systematics. The basal position within the euryalid clade supported by our study sheds a fundamentally new light on the *Aspiduriella* group.

Although the general morphology of *A. scutellata* is superficially atypical of euryalids, some details clearly point towards euryalid affinities, in particular the short adoral shields meeting over their entire length, as well as the disc granules. Even the vertebrae show some euryalid affinities with their nearly hourglass-shaped zygocondyles that differ only from euryalid equivalents in having a tiny zygosphene. The most revealing piece of evidence, however, is provided by the lateral arm plates, in particular the unornamented outer surface and the shape of the spine articulations. These new microstructural insights into the *Aspiduriella* group and their phylogenetic implications allow tracing back the evolutionary history of euryalids into the Middle Triassic, in line with a latest Paleozoic to early Mesozoic radiation of crown-group ophiuroids^14^.

*Aspiduriella streichani* (Kutscher, 1987)

Figure 4

*1987 *Aspidura streichani* Kutscher, p. 703, Fig. 1–8.

1998 *Aspiduriella streichani* (Kutscher): Bolette, p. 401.

Material examined: GZG.INV.90016 - GZG.INV.90019.

#### Description

**Figure 4:**
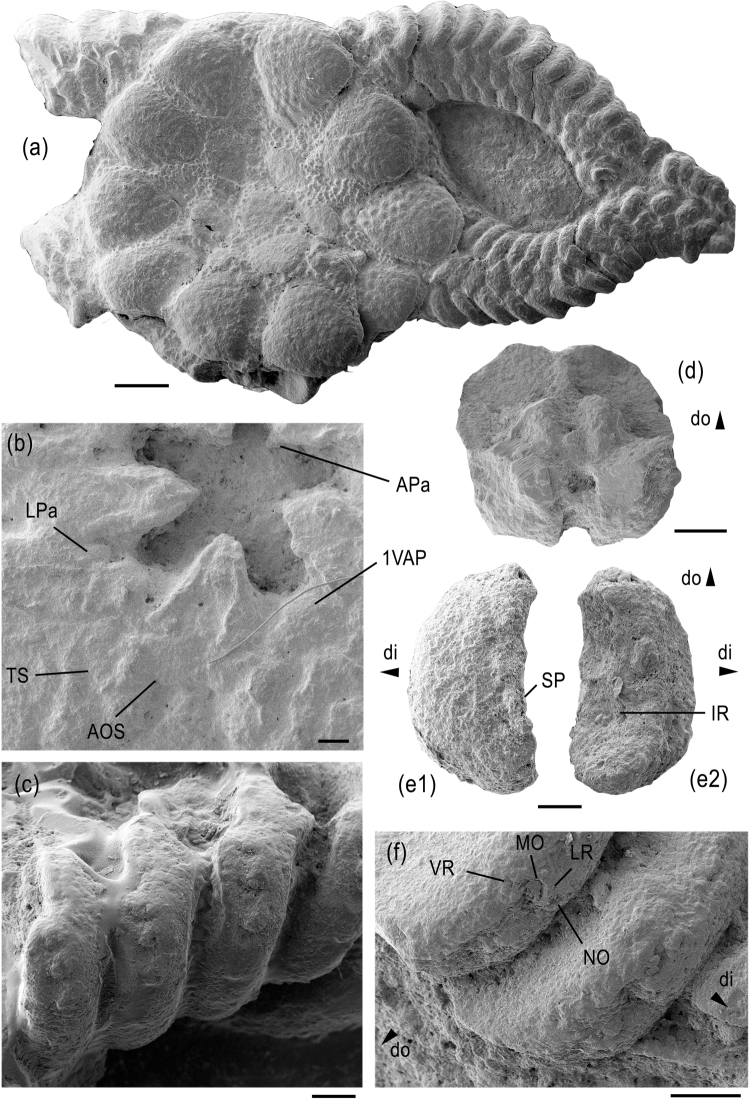
*Aspiduriella streichani* from the Middle Triassic of Rüdersdorf, Germany. (**a**) GZG.INV.90016, articulated skeleton preserving two arms and proximal segments of a third, exposing the dorsal side. (**b**) GZG.INV.90017, detail of oral plating of an articulated skeleton. (**c**) GZG.INV.90018, detail of proximal arm segments of an articulated skeleton in ventro-lateral view. (**d**) GZG.INV.90018, isolated vertebra in distal view. (**e**) GZG.INV.90019, isolated lateral arm plate in external (e1) and internal (e2) views. (**f**) GZG.INV.90018, proximal lateral arm plates of an articulated skeleton showing detail of spine articulations in lateral view. Abbreviations: AOS: adoral shield; APa: apical papilla; di: distal; do: dorsal; IR: ridge on the inner side of the lateral arm plate; LPa: lateral oral papilla; LR: lip-shaped ridge; MO: muscle opening; NO: nerve opening; SP: spur; TS: tentacle scale; VR: vertical ridge proximally bordering muscle opening; 1VAP: first ventral arm plate. Scale bars equal 1 mm (**a**) and 0.2 mm (**b**–**f**).

Medium-sized species (disc diameter of examined specimens between 5 and 7 mm), disc round (Fig. 4a), dorsal side dominated by nearly continuous ring of radial shields; radial primary plates large, round, in contact with nearly equal-sized central primary plate but not forming a continuous ring; radial shields (Fig. 4a) conspicuously large and thick, equalling more than half of the disc radius, not covered by granules but with coarse tubercles, rounded isosceles triangular with weakly convex lateral edges; radial shields and radial primary plates separated by few tiny, round scales covered by granules. Ventral disc interradii with small, rounded hexagonal oral shield, slightly longer than wide, proximally, laterally and distally bordered by tiny scales densely covered by granules; genital slits short, extending from adoral shields to almost half of the interradius, no appendages other than regular disc granules; adoral shields (Fig. 4b) almost as long as wide, lozenge-shaped, in contact over their entire length, boundaries covered by granules; jaws relatively short, conspicuously slender and pointed; five small, leaf- to block-like lateral oral papillae (Fig. 4b) forming a continuous row, increasing in size from the jaw tip to the adoral shield; second oral tentacle pore entering mouth slit via shallow embayment; first ventral arm plate triangular with convex proximal tip projecting into mouth slit and lateral edges bearing two block-like papillae similar in shape to median lateral oral papillae but slightly larger; single, very small, short, conical and pointed apical oral papilla.

Arms short (Fig. 4a), broad at base, rapidly tapering; not branching and not covered by granules except possibly for the plate boundaries on the ventral side of the arm segments incorporated into the disc; dorsal arm plates (Fig. 4a) medium-sized, the proximal ones wider than long, with right proximal angle, acute lateral angles and obtuse distal angle with weakly concave latero-distal edges; dorsal arm plates in contact up to median arm segments; lateral arm plates (Fig. 4c,e) thick, bulging, crescent-shaped, the proximal ones much higher than long, with pointed dorso-proximal tip and enlarged, ventro-proximalwards pointing ventral portion with oblique, concave ventral edge; outer surface stereom with simple trabecular intersections, devoid of ornamentation except for a series of at least six small, poorly defined, weakly prominent spurs lining the central part of the proximal edge; row of spurs distally bordered by slightly depressed central part of the lateral arm plate; up to five spine articulations (Fig. 4f) sunken into distal edge of lateral arm plates, equal-sized except for a slightly smaller ventralmost one, and with dorsalwards increasing distance between the spine articulations; muscle opening of spine articulations proximally bordered by arched, non-denticulate vertical ridge, and distally separated from nearly equal-sized nerve opening by short, thick, lip-shaped and moderately prominent ridge; inner side of lateral arm plates with single, slender, vertical ridge composed of the same stereom as remaining inner side, zig-zag-like with three kinks (Supplementary Fig. 4b); arm spines parallel to the arm axis, as long as one quarter the length of an arm segment, smooth, laterally flattened and with blunt tip; no hooks or modified spines discernible; ventral arm plates moderately large, in contact up to median arm segments, nearly diamond-shaped with obtuse distal angle, concave latero-proximal edges lined by a prominent, slender ridge, and acute proximal angle; tentacle openings large, developed as elongate between-plate openings at an angle to the arm axis, covered by at least six flat, elongate tentacle scales, three at the lateral arm plate and another three at the ventral arm plate; vertebrae (Fig. 4d) with a small zygosphene between two large, parallel and almost hourglass-shaped zygocondyles.

#### Remarks

The euryalid affinities of the *Aspiduriella* group were first hinted at on the basis of *Aspiduriella streichani*^14,22^. Indeed, *A. streichani* displays the diagnostic microstructural features in the most conspicuous way, as compared to its congeners, which is at least in part due to its comparatively large size. Another factor, however, might be that *A. streichani* seems to be less strongly paedomorphic than its congeners, as indicated by the greater number of disc scales surrounding and in part separating the primary plates, by the higher lateral arm plates and by the ventral and dorsal arm plates that abut in proximal and median arm segments^25^, in line with preliminary observations that paedomorphosis may conceal morphological traits of phylogenetic importance^15^.

The differentiation between *A. streichani* and its congeners including *A. scutellata* was convincingly discussed in the original species description^26^. The aim of the present re-description was to unlock previously overlooked characters of phylogenetic relevance, with special emphasis on lateral arm plates. It turned out that, in terms of lateral arm plate morphology, *A. streichani* bridges the morphological gap between *Aspiduriella* and *Melusinaster* gen. nov. Especially the height/length ratio of the lateral arm plates, the large, strongly protruding ventral portion and the shape of the ridge on the inner side are strongly reminiscent of *Melusinaster* gen. nov., but unknown in any other species of *Aspiduriella*. Whether or not, on these grounds, *A. streichani* should be separated on genus level from its current congeners remains to be decided in a more exhaustive revision of the *Aspiduriella* group. Within the limited scope of the present study, we consider *A. streichani* as the least paedomorphic representative of the *Aspiduriella* group within the early euryalid stem.

Genus *Melusinaster* nov.

urn:lsid:zoobank.org:act:C70BEA51-C832-4B88-9A7D-310370DC8D00

Type species: *Melusinaster alissawhitegluzae* sp. nov., by present designation

#### Diagnosis

Small ophiuroid with high to very high, strongly arched, crescent-shaped lateral arm plates; outer surface of lateral arm plates devoid of tubercles, spurs or any other ornamentation; ventral portion of lateral arm plates large and strongly protruding; spine articulations composed of large, vertical, slit-like muscle and nerve openings, muscle opening proximally and distally bordered by large, well-defined, vertical, strongly protruding and lip-shaped ridges; inner side of lateral arm plate with well-defined, zig-zag-like ridge; vertebrae with deep and uncovered ventral neural canal; distal articulation face of vertebrae with large, hourglass-shaped, nearly parallel zygocondyles composed of coarsely meshed stereom abaxially and densely meshed stereom adaxially; lateral saddles of vertebrae with area of coarsely meshed stereom corresponding to ridge on inner side of lateral arm plates and suggesting almost complete lateral encompassing of arms by lateral arm plates.

#### Etymology

The name refers to Melusina, a mythical figure of European folklore. According to the Luxembourg version of the legend, Count Sigefroid, founder of Luxembourg City, was enchanted by the captivating singing of Melusina and proposed to her. On her terms of marriage, however, she requested absolute privacy every Saturday. Unable to resist temptation, Sigefroid spied on her on one of the forbidden days and discovered she was a mermaid. Melusina, horrified, vanished into the cliff, never to be seen again by Sigefroid. The direct implication of the legend within the context of the fossils described herein is that nothing is what it seems to be at first sight.

Gender: masculine.

Other species included: *Melusinaster arcusinimicus* sp. nov.

*Melusinaster alissawhitegluzae* sp. nov.

Figure 5a–d

urn:lsid:zoobank.org:act:AA407FFB-24B0-4781-A336-79D9A092EA1A

1996 *Sigsbeia*? *lunaris* (Hess, 1962): Kutscher, p. 12, pl. 3–4.

2003 *Sigsbeia*? *lunaris* (Hess, 1962): Kutscher & Villier, p. 189, fig. 6(7–8), fig. 7(1–2).

2014 unnamed stem euryalid: O’Hara *et al*., Fig. 2K-L.

2015 unnamed euryalid: Thuy, Fig. 1A–C.

#### Etymology

Species name chosen by BT to honour Alissa White-Gluz, singer of death metal band Arch Enemy, for being an inspiring person, and to pay tribute to the intensity, authenticity and passion that she conveys in her powerful vocals.

Holotype: MnhnL OPH028

Type locality and stratum: field in the Seweckenberge area southeast of Quedlinburg, Germany; clay bed with reworked ammonite casts dated as latest Toarcian (Torulosum Subzone, Aalensis Zone), Lower Jurassic (ca. 174 million years).

Paratypes: MnhnL OPH029 - OPH031

Other material studied: MnhnL OPH032 (26 dissociated lateral arm plates and 22 vertebrae)

#### Diagnosis

Species of *Melusinaster* gen. nov. with very high lateral arm plates, proximal ones 2.5 times higher than long; with strongly protruding, spoon-like ventral portion; up to eight spine articulations in proximal lateral arm plates, the ventral five with merged proximal and distal ridges and the three dorsal ones separated and becoming increasingly oblique; ridge on inner side of proximal lateral arm plates with three kinks.

#### Description of holotype

**Figure 5:**
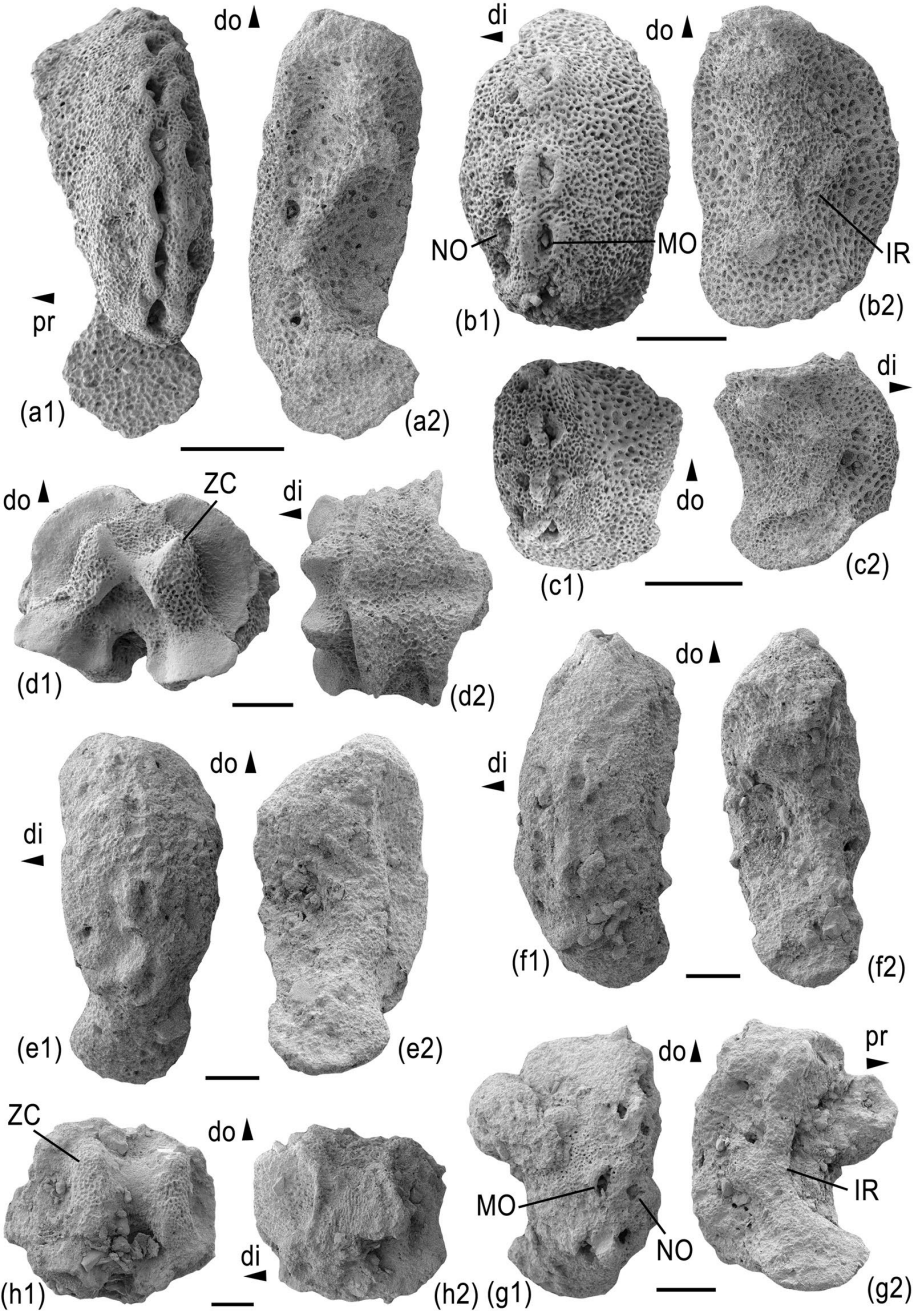
*Melusinaster alissawhitegluzae* gen. et sp. nov., from the Lower Jurassic (Torulosum Subzone, Aalensis Zone, uppermost Toarcian) of Quedlinburg, Germany. (**a**) MnhnL OPH028 (holotype), dissociated proximal lateral arm plate in external (a1) and internal (a2) views. (**b**) MnhnL OPH029 (paratype), dissociated median lateral arm plate in external (b1) and internal (b2) views. (**c**) MnhnL OPH030 (paratype), dissociated distal lateral arm plate in external (c1) and internal (c2) views. (**d**) MnhnL OPH031 (paratype), dissociated proximal vertebra in distal (d1) and dorsal (d2) views. *Melusinaster arcusinimicus* gen. et sp. nov., from the Middle Jurassic (Humphriesianum Zone, lower Bajocian) of Rumelange, Luxembourg. (**e**) MnhnL OPH033 (holotype), dissociated proximal lateral arm plate in external (e1) and internal (e2) views. (**f**) MnhnL OPH034 (paratype), dissociated proximal lateral arm plate in external (f1) and internal (f2) views. (**g**) MnhnL OPH035 (paratype), dissociated median lateral arm plate in external (g1) and internal (g2) views. (**h**) MnhnL OPH036 (paratype), dissociated proximal vertebra in distal (h1) and lateral (h2) views. Abbreviations: di: distal; do: dorsal; IR: ridge on the inner side of the lateral arm plate; MO: muscle opening; NO: nerve opening; pr: proximal; ZC: zygocondyle. Scale bars equal 0.1 mm.

MnhnL OPH028 (Fig. 5a) is a dissociated proximal lateral arm plate approximately 2.5 times higher than long; dorsal tip of lateral arm plate pointed, proximal edge straight, distal edge weakly convex; ventral portion strongly protruding ventro-proximalwards, with enlarged, spoon-like lower part and separated from remaining lateral arm plate by a constriction; trabecular intersections of outer surface stereom not enlarged or transformed in any way; outer surface of lateral arm plate devoid of spurs, depressions or any other ornamentation; dorso-proximal part of outer surface slightly lower than remaining plate and with slightly more coarsely meshed stereom; eight large, equal-sized spine articulations in a continuous row on the slightly bulging central part of the lateral arm plate; all spine articulations composed of large, nearly equal-sized, slit-like muscle and nerve openings; muscle openings proximally bordered by a well-defined, prominent, slender and arched ridge, and distally bordered by a similarly well-defined and arched, yet wider and more prominent ridge; the five ventral spine articulations vertical, with proximal and distal ridges merged; the three dorsal spine articulations separated by increasingly large gaps and gradually becoming increasingly more inclined with respect to the five vertical ventral spine articulations; ridges of the three dorsal spine articulations separated; inner side of lateral arm plate with large, moderately well-defined, slender, strongly prominent ridge composed of four parts connected by angular kinks resulting in a zig-zag-like shape; ventralmost part of ridge poorly defined, gradually merging into ventral edge of lateral arm plate; distal edge of lateral arm plate lined by a shallow, poorly defined furrow.

#### Paratype supplements

MnhnL OPH029 (Fig. 5b) is a dissociated median lateral arm plate almost two times higher than long; all edges gently convex, resulting in generally rounded aspect of lateral arm plate; outer surface stereom as in holotype; ventral portion much smaller than in holotype and only weakly protruding; four spine articulations similar to those of holotype, with dorsalward increase in size of gaps between the spine articulations; dorsalmost spine articulation oblique; ridge on inner side of lateral arm plate poorly defined, wider than ridge of holotype and only with two kinks; with spots of more densely meshed stereom on dorsal and ventral tips and on the distal tip of the dorsal kink; no furrow discernible.

MnhnL OPH030 (Fig. 5c) is a dissociated distal lateral arm plate slightly higher than long, otherwise very similar to paratype MnhnL OPH029; spine articulations on more strongly bulging distal portion of lateral arm plate; ridge on inner side as in paratype MnhnL OPH029 but only with one kink.

MnhnL OPH031 (Fig. 5d) is a dissociated proximal to median vertebra, dorso-distal muscle fossae moderately large, evenly round; ventro-distal muscle fossae robust, fan-shaped, protruding, distalwards convex; dorsal side with coarsely meshed stereom and with poorly defined, shallow longitudinal groove; ventral neural canal deep and uncovered; distal articulation face with hourglass-shaped, near-parallel zygocondyles composed of coarsely meshed stereom abaxially and densely meshed stereom adaxially.

Remarks: The lateral arm plates and vertebrae described above were already published by Kutscher^18^ who identified them as *Sigsbeia*? *lunaris* (Hess, 1962). Later, Kutscher and Villier^27^ illustrated similar and most probably conspecific lateral arm plates from the Lower Jurassic of France, again under the name of *Sigsbeia*? *lunaris*. Examination of the type material of *S*.? *lunaris* housed in the collections of the Natural History Museum Basel, however, has revealed fundamental differences which preclude assignment to the same species or even the same genus.

**Figure 6:**
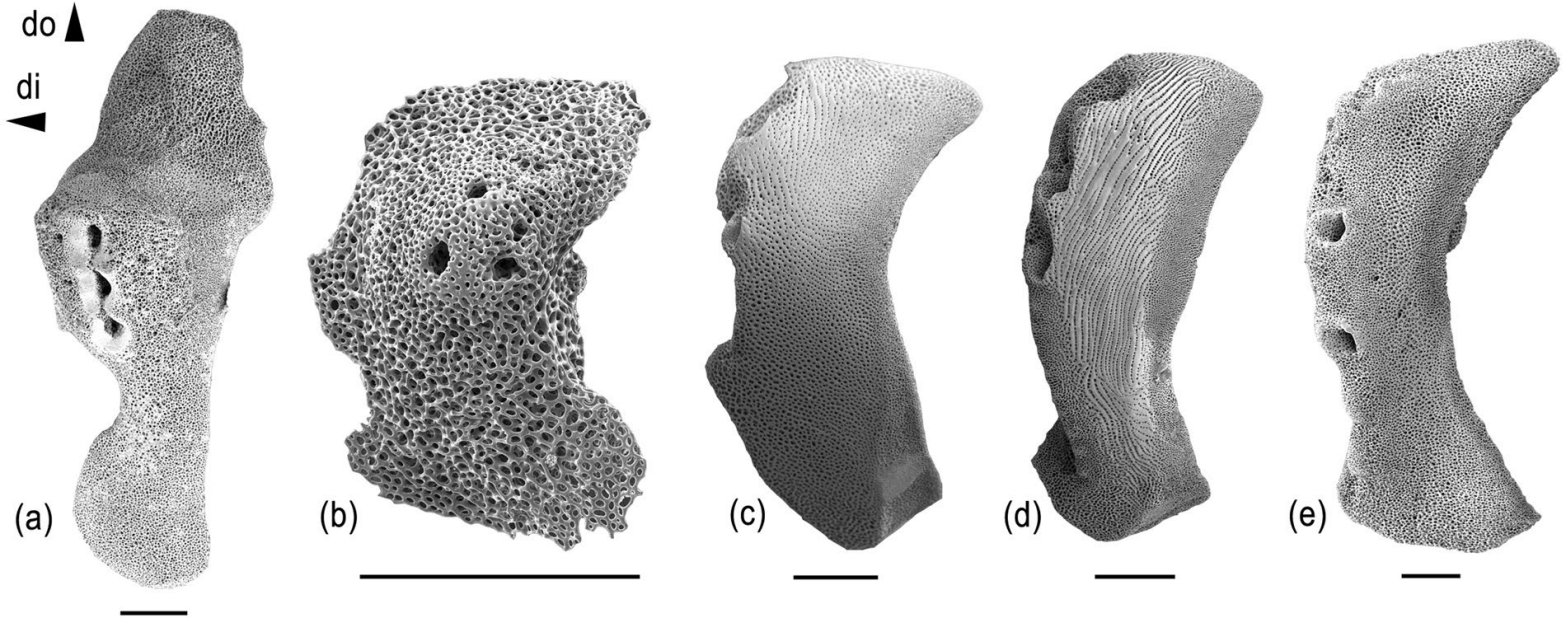
Lateral arm plates of Recent euryalid and ophiurid ophiuroids. (**a**) SMNH-131549, *Gorgonocephalus caputmedusae*. (**b**) *Astrodia abyssicola*, image courtesy of Masanori Okanishi. (**c**) MnhnL OPH038, *Ophiura flagellata*. (**d**) SMNH-121756, *Ophioperla koehleri*. (**e**) *Ophiosparte gigas*. Abbreviations: di: distal; do: dorsal Scale bars equal 0.5 mm.

The lateral arm plates described above, along with their probable equivalents published by Kutscher and Villier^27^, thus represent a previously unrecognized type of fossil lateral arm plates. We here assign it to the new taxon *Melusinaster alissawhitegluzae* gen. et sp. nov., because it stands out against other types of fossil lateral arm plates with respect to spine articulation morphology, which, at the same time, provides the most revealing piece of evidence for its true systematic position: strikingly similar spine articulations are found in lateral arm plates of modern euryalids such as the gorgonocephalid *Gorgonocephalus* (Fig. 6a). The shape of the lateral arm plates and the completely smooth outer surface stereom are also strongly reminiscent of those found in modern euryalids, especially the asteronychid *Astrodia* (Fig. 6b).

The euryalid affinities of *Melusinaster alissawhitegluzae* gen. et sp. nov. were anticipated by O’Hara *et al*.^14^ and Thuy^22^ and are here corroborated by an in-depth morphological analysis. In our phylogenetic estimate, this new taxon emerges at the base of the modern euryalids, albeit in a more derived position than *Aspiduriella*. This aligns perfectly well with the stratigraphical and morphological transition from the Triassic *Aspiduriella* to the modern euryalids via the Jurassic *Melusinaster* gen. nov.

The exact position of *Melusinaster* gen. nov. within the euryalid clade cannot be resolved with the currently available evidence. A systematic search for other fossil euryalids and a more exhaustive sampling of modern euryalids with emphasis on phylogenetically relevant morphological characters will enable more conclusive insights in this respect. We anticipate, however, that *Melusinaster* gen. nov. cannot be accommodated in one of the three living euryalid families, especially because the lateral arm plates of the new genus were in a lateral position, as in *Aspiduriella*, encompassing a greater portion of the arm, rather than reduced to a small part of the ventral side of the arms as in modern euryalids.

*Melusinaster alissawhitegluzae* gen. et sp. nov. differs from its congener *M. arcusinimicus* sp. nov. in having higher and more crescent-shaped lateral arm plates with a pointed dorsal edge and with a greater number of spine articulations. We chose *M. alissawhitegluzae* sp. nov. as the type species of the genus because it is known from extensive and very well-preserved material.

With respect to the type stratum of *M. alissawhitegluzae* gen. et sp. nov., relevant details were supplied by Kutscher^18^. It should be added, however, that the associated ammonite fragment identified as *Pachylytoceras torulosum* Schubler, 1831 indicates the latest Toarcian Torulosum Subzone within the Aalensis Zone^28^, in agreement with the ostracod evidence, and not Aalenian as stated by Kutscher^18^. The age of *M. alissawhitegluzae* gen. et sp. nov. is thus precisely constrained.

*Melusinaster arcusinimicus* sp. nov.

Figure 5e–h

urn:lsid:zoobank.org:act:51A6B3A2-73A2-4B9E-A5D9-B01A6FE500B7

#### Etymology

Species name chosen by BT and corresponding to the Latin translation of Arch Enemy, Swedish death metal band, for producing some of the heaviest melodic death metal songs ever, and in particular for having written masterpieces such as ‘We will rise’ and ‘Reason to believe’.

Holotype: MnhnL OPH033

Type locality and stratum: disused limestone quarry “Wäisskaul” at Rumelange, Luxembourg; greenish-grey clay pockets within coral reef limestone dated as early Bajocian (Humphriesianum Zone), Middle Jurassic (ca. 167 million years).

Paratypes: MnhnL OPH034 - OPH036

Other material studied: MnhnL OPH037 (2 dissociated lateral arm plates and 2 vertebrae)

#### Diagnosis

Species of *Melusinaster* gen. nov. with high lateral arm plates, proximal ones slightly more than two times higher than long; with oblique dorsal edge and strongly protruding, spoon-like ventral portion; three spine articulations in proximal lateral arm plates, in some lateral arm plates associated with a fourth slightly larger and prominent one near the dorsal tip of the plate; ridge on inner side of proximal lateral arm plates with two kinks, the dorsal one with a distalwards pointing protrusion.

#### Description of holotype

MnhnL OPH033 (Fig. 5e) is a dissociated proximal lateral arm plate slightly more than two times higher than long; dorsal edge of lateral arm plate oblique, proximal and distal edges evenly convex; ventral portion strongly protruding ventro-proximalwards, with lower part enlarged and separated from remaining lateral arm plate by a constriction; trabecular intersections of outer surface stereom simple, not enlarged or transformed in any way; outer surface of lateral arm plate devoid of spurs, depressions or any other ornamentation; three large, equal-sized spine articulations in a continuous vertical row in the middle of the lateral arm plate; all spine articulations composed of large, nearly equal-sized, slit-like muscle and nerve openings; muscle openings proximally bordered by a well-defined, prominent, slender and arched ridge, and distally bordered by a similarly well-defined and arched but wider and more strongly prominent ridge; the two dorsal spine articulations vertical, the ventralmost one oblique; gap between middle and dorsal spine articulation largest; inner side of lateral arm plate with large, moderately well-defined, strongly prominent ridge composed of three parts all connected by angular kinks: a strongly oblique ventro-proximalwards pointing ventral part, a less strongly oblique ventro-proximalwards pointing central part, and a slightly oblique dorso-proximalwards pointing dorsal part; distal edge of lateral arm plate lined by a shallow, poorly defined furrow.

#### Paratype supplements

MnhnL OPH034 (Fig. 5f) is a dissociated proximal lateral arm plate nearly two times higher than long; general morphology as in holotype except for less pronounced constriction between main part and ventral portion; four spine articulations, the ventral three as in holotype, and the fourth near the dorsal tip of the lateral arm plate, larger than the others and strongly prominent; inner side of lateral arm plate as in holotype.

MnhnL OPH035 (Fig. 5g) is a dissociated median lateral arm plate slightly more than 1.5 times higher than long; moderately well preserved with skeletal debris attached to outer proximal edge; general morphology as in holotype except for evenly convex rather than oblique dorsal edge; three spine articulations as in holotype; ridge on inner side of lateral arm plate better preserved than in holotype, sharply defined, composed of the same three parts as in holotype, ventral edge of ventral part fading into ventral plate edge, and kink between central and dorsal parts with distalwards pointing protrusion; dorsal tip of dorsal ridge part sharply defined.

MnhnL OPH036 (Fig. 5h) is a dissociated proximal to median vertebra, moderately well preserved, albeit cluttered with skeletal debris; dorso-distal muscle fossae small, evenly round; ventro-distal muscle fossae robust, fan-shaped, protruding, distalwards convex; dorsal side with poorly defined, shallow longitudinal groove; ventral neural canal deep and uncovered; distal articulation face with conspicuously large, hourglass-shaped, nearly parallel zygocondyles composed of coarsely meshed stereom abaxially and densely meshed stereom adaxially; lateral saddles wide, distally and proximally sharply bordered by edges of muscle fossae, with poorly defined, slightly prominent area composed of more coarsely meshed stereom and corresponding to shape of ridge on inner side of the lateral arm plates, documenting a near-complete lateral encompassing of the arms by the lateral arm plates.

#### Remarks

Lateral arm plates and vertebrae from the same sample as those described above were briefly mentioned by Thuy^22^ in a preliminary note on the early euryalid fossil record. We here describe these remains in detail and assign them to the new genus *Melusinaster*, given its overall similarity with the type species *M. alissawhitegluzae* sp. nov. with respect to the shape and outer surface ornamentation of the lateral arm plates and the spine articulation morphology. The generally lower height/length ratio of the lateral arm plates, the oblique rather than pointed dorsal edge, the lower number and different arrangement of the spine articulations and the shape of the ridge on the inner side of the lateral arm plates, however, favor separation at the species level.

## Discussion

In our phylogenetic analyses, *Aspiduriella* and *Melusinaster* gen. nov. invariably emerge as members of the euryophiurids. Relationships within this clade, however, appear strongly influenced by *Ophiosparte*. The unusual morphology of this Antarctic genus has challenged traditional classification concepts and deceptively suggested an ophiacanthid^29,30^ or ophiomyxid^31^ relationship. Recent advances in the understanding of ophiuroid micromorphology have provided evidence of ophiurid affinities^10,17^ that have subsequently been corroborated by phylogenetic estimates^15,16^. Interestingly, Martynov^10^ considered *Ophiosparte* to be an archaic intermediate between the Ophiuridae and the Euryalida. While molecular evidence clearly unmasked *Ophiosparte* as a relatively derived member of the ophiurid family Ophiopyrgidae^2^, morphology-based phylogenies provided support for closer ties with the euryalids^15^.

We carefully reassessed the morphology of *Ophiosparte* and singled out three lateral arm plate characters that seemed among the most euryalid-like. In fact, in *Ophiosparte*, the outer surface stereom of the lateral arm plates lacks enlarged trabecular intersections, tubercles, striations, spurs or any other elements of ornamentation, setting the genus apart from other ophiopyrgids. This lack of ornamentation is either ancestral, and thus shared with the euryalids, or the result of a secondary loss of previously existing ornamentation.

In the most recent molecular phylogeny of the Ophiuroidea^16^, *Ophiosparte* is nested within the Ophiopyrgidae in a close relationship with *Ophiura flagellata* (Lyman, 1878) (Fig. 6c) and *Ophioperla koehleri* (Bell, 1908) (Fig. 6d). In both species, the lateral arm plates have a well-developed vertical striation and a spur on the ventro-proximal tip, strongly favouring secondary loss to explain the absence of these features in *Ophiosparte* (Fig. 6e). When this assumption is taken into account in our phylogenetic estimate, i.e. when the absence of lateral arm plate ornamentation is scored differently in euryalids versus *Ophiosparte*, the seemingly close ties between the two fall apart, suggesting that *Ophiosparte* is, in fact, a derived ophiopyrgid with independently acquired euryalid-like features. Based on this conclusion, we felt confident to exclude *Ophiosparte* from our final estimate in an attempt to minimize noise caused by homoplasies. The decision to exclude *Ophiosparte* is corroborated by an explorative Bayesian estimate of the matrix used by Thuy and Stöhr^15^ minus *Ophiosparte*: the euryalids emerge as sister clade to the two remaining extant ophiurids (*Ophiura* and *Ophiocten*), in agreement with molecular evidence^14,16^, rather than at the tip of a nested ophiurid succession. The low node support for the split between the Ophiurida and Euryalida is possibly related to *Aspiduriella* and *Melusinaster* gen. nov. representing morphologically transitional species, sharing characters with both clades.

Our phylogenetic estimate successfully includes a fossil taxon known exclusively from dissociated skeletal ossicles, in the present case lateral arm plates and vertebrae. Thus, not only do we corroborate that ophiuroid microfossil records can indeed be integrated in phylogenies^15^ but also document that they can add significant value by bridging morphological gaps in spite of their incompleteness. The evolutionary history of the euryalids is intimately bound to the origin of asterozoan arm branching since they are the only asterozoans known to date to include taxa with branched arms. With respect to skeletal morphology, taxa with branching arms have modified vertebrae at the arm bifurcations, showing double distal articulation faces. In the fossil record, the oldest such branching vertebrae are known from the Neogene^6,7,12,32^. All known species of *Aspiduriella* have simple arms, and vertebrae assignable to *Melusinaster* gen. nov. all have simple distal articulation faces. Thus, the fossil record is only helpful in so far as it suggests a Late Jurassic or younger origin of arm branching in euryalids. In the phylogeny outlined by O’Hara *et al*.^16^, genera with branching arms are found only in the sister clades Gorgonocephalidae and Euryalidae. Their sister clade Asteronychidae, in contrast, lacks genera with branching arms. A single origin for branching arms seems the most parsimonious hypothesis, also because they are unknown in any other asterozoan group, implying that it coincides with the Gorgonocephalidae-Euryalidae node. Inferred from the node dates suggested by O’Hara *et al*.^16^, the oldest branching vertebrae are thus to be expected in Lower Cretaceous strata.

## Conclusion

We explore the evolutionary history of euryalids based on a reassessment of previously known fossil ophiuroids and newly collected material. A morphology-based phylogenetic estimate identifies the Triassic genus *Aspiduriella* as a member of the euryalid clade. *Aspiduriella* superficially resembles the modern ophiurid sister clades of the euryalids but presents some microstructural features pertaining to the lateral arm plates, the spine articulations and the vertebrae that clearly point to a euryalid ancestry. We furthermore describe the new Jurassic genus *Melusinaster* based on dissociated lateral arm plates and vertebrae as an early euryalid morphologically intermediate between *Aspiduriella* and the extant euryalids. Our analysis fills a major gap in the fossil record of the euryalids, substantiating a predicted phylogenetic ghost lineage of the clade into the Triassic. It also sets a robust phylogenetic framework to trace the morphological transition from ophiurid-like ancestors to the typical modern euryalid, explaining the conspicuous morphological disparity between members of the extant sister clades Ophiurida and Euryalida (Fig. 2). Finally, our study presents the first morphological phylogeny that successfully combines data from completely preserved skeletons and data extracted from isolated microfossils in order to bridge morphological and stratigraphic gaps between the sampled taxa.

## Electronic supplementary material

Supplementary Info

Supplementary Dataset 2
